# Mechanisms and Implications of Age-Related Changes in the Liver: Nonalcoholic Fatty Liver Disease in the Elderly

**DOI:** 10.1155/2011/831536

**Published:** 2011-09-12

**Authors:** Lay Gan, Shivakumar Chitturi, Geoffrey C. Farrell

**Affiliations:** Liver Research Group, Gastroenterology and Hepatology Unit, The Canberra Hospital, Australian National University Medical School, Yamba Drive, Garran, ACT 2605, Australia

## Abstract

Nonalcoholic fatty liver disease (NAFLD) is hepatic steatosis associated with metabolic abnormalities such as overweight/central obesity, insulin resistance, type 2 diabetes (T2D), and dyslipidemia. NAFLD is becoming the most common liver disease in contemporary society, with the highest prevalence in those over 60 years. NAFLD pathology ranges from simple steatosis to a necroinflammatory fibrosing disorder called steatohepatitis (SH), the latter associated with high risk of developing cirrhosis, often occuring in the seventh to ninth decades of life. While the main health implications of NAFLD are increased risk of developing T2D, cardiovascular diseases, and common cancers, there is substantantially increased standardized mortality, and deaths from decompensated cirrhosis and hepatocellular carcinoma (HCC). Little is known about the interactive effects of ageing and NAFLD, with most studies focusing on the younger population. This paper summarises the epidemiology, pathogenesis, and clinical course of NAFLD, with particular attention to persons over age 60 years. An approach to the management of NASH and its complications in the elderly, will also be presented here.

## 1. Introduction

In 1980, Ludwig and colleagues introduced the concept of nonalcoholic steatohepatitis (NASH) to describe liver histologic changes resembling alcoholic hepatitis in individuals without significant alcohol intake [1]. NASH is now conceptualized as part of a pathological spectrum of a fatty liver disorders caused by metabolic factors and referred to collectively as nonalcoholic fatty liver disease (NAFLD). The mildest form of NAFLD is simple steatosis, characterised by hepatic fat (triglyceride, TG) accumulation alone. In NASH, hepatic necroinflammatory changes are also present and a characteristic perisinusoidal pattern of liver fibrosis is common. Up to 25% of NAFLD patients have NASH, and in perhaps one third of such cases there is slowly progressive liver fibrosis leading to cirrhosis. Primary liver cancer (hepatocellular carcinoma, HCC) is now a recognized complication of NAFLD, usually but not always after development of cirrhosis [2–5] 

Ludwig's descriptions of NASH suggested higher prevalence in women, particularly in those who are obese and have type 2 diabetes (T2D) [1]. Over the last decade, community-based studies have found male predominance of NAFLD from the paediatric population [6] up to fifth decade of life in adults. After age 60 years, however, females overtake their male counterparts in prevalence of NAFLD [7], an age and gender distribution that resembles that of cardiovascular disease. This is not surprising given similar risk factors for NAFLD and cardiovascular diseases. 

Most cases of NAFLD occur in overweight or obese individuals, and there are particular strong links to central obesity, T2D, atherogenic dyslipidemia, and hypertension, each of which are elements of metabolic syndrome [8, 9]. NAFLD can now be regarded as the hepatic manifestation of the metabolic syndrome, although it is not yet included as a definitional component. The links between NAFLD and metabolic syndrome/prediabetes/T2D seem likely to reflect the operation of shared pathogenic factors, as we and others have reviewed [10–12]. Thus, the presence of fatty liver is a strong, independent predictor for the future development of metabolic syndrome [13–15], T2D, and cardiovascular events. A minority of cases of fatty liver (not due to alcohol) are secondary to specific etiologic agents such as drugs or occur in well-defined settings (jejuno-ileal bypass, total parenteral nutrition); they are not regarded as NAFLD (which infers a metabolic etiology) and will be not discussed further here [12, 16, 17].

In this paper, we will first review the epidemiology and pathophysiology of NAFLD, with particular focus on age-related aspects. We will then examine risk factors for developing progressive liver injury and how known age-related changes in liver biology might contribute to severity of the disease among older people. The paper will conclude with some comments about management strategies that may be applicable to older individuals with fatty liver. 

## 2. Epidemiology

### 2.1. Diagnosis

The current diagnosis of NAFLD is based on detection of hepatic steatosis by liver biopsy or imaging, exclusion of other liver diseases, particularly alcohol and hepatitis C, and recognition of metabolic risk factors. The true prevalence of NASH, the most clinically relevant subset of patients with NAFLD, has been difficult to establish because this is a histologic diagnosis. The diagnosis of NAFLD often comes to light because of detection of abnormal liver tests, particularly raised serum alanine aminotransferase (ALT) and gamma-glutamyl transpeptidase (GGT) and serum ferritin.

All noninvasive methods for diagnosis of NAFLD have limitations—computerised tomography (CT) scan and hepatic ultrasonography (US) are relatively insensitive; they can only detect moderate to severe steatosis [5, 18]. Serum ALT increase is not only insensitive, but also nonspecific; values may remain within the normal range in up to 80% of patients with biopsy-proven NAFLD and up to 30% of NASH [19]. Similarly, serum ferritin is a nonspecific inflammatory marker, but while it is not specific for diagnosis of NAFLD, once the diagnosis has been established, serum ferritin has been shown to be significantly more elevated in patients with NASH compared with those with simple steatosis (SS) [20]. 

The “gold standard” of NASH diagnosis is liver biopsy. However, apart from being an unpleasant and occasionally dangerous test, it is subject to sampling error and observer differences in histopathologic interpretation. Further, reported studies of NASH prevalence may suffer from selection and ascertainment biases as liver biopsies were often performed in specific groups like the morbidly obese and diabetics or among highly selected subjects enrolled in clinical trials [21]. 

In the last decade, transient elastography (TE) has been developed as a safe noninvasive alternative to liver biopsy for assessing liver fibrosis. This technique has been validated across different liver diseases, including NAFLD [22]. A recent study of 246 biopsy-proven NAFLD patients from Hong Kong and France suggested decreasing ability to successfully obtain liver stiffness measurements (LSM) with increasing BMI; measurement was 97% successful with BMI < 30 kg/m^2^, dropping to only 75% in obese patients, though this did not reach statistical significance [23]. However, the recent introduction of the “obese” probe allows LSM to be successfully increased from 45% to 76% in the morbidly obese (mean BMI > 40 kg/m^2^) [24]. In the elderly population, TE is a promising noninvasive method of quantifying liver fibrosis in NAFLD.

### 2.2. Community Prevalence of NAFLD and NASH: Rising Global Prevalence

Depending on the screening tool and when the study was performed, estimated community prevalence of NAFLD has ranged from 2.8% to 46% [25–27]. In the Dallas Heart Study, Browning et al. used proton magnetic resonance spectroscopy (MRS) to quantify hepatic triglyceride (TG) content of 3 major ethnic groups. Hepatic steatosis (defined as greater than 5.5% TG content) was noted in 31% overall, with significant ethnic variation—45% in hispanics, 33% in whites, and 24% in blacks [28]. In Japan, Korea, Taiwan, and China, the use of ultrasonography in community studies has found rates between 5 and 40% [29], with an increasing prevalence over the last 20 years [29, 30]. A recent study in predominantly middle-aged American employees or outpatients (without known liver disease) found that the prevalence of steatosis by ultrasonography was 47%; in this study, NASH was confirmed histologically in 12% of the total cohort or 30% of the ultrasound-positive subgroup [27]. Another recent analysis of nearly 40,000 patients from three cycles of the National Health and Nutritional Examination Survey (NHANES) conducted between 1988 and 2008 showed rising prevalence of NAFLD from approximately 5% in the 1988–1994 cohort to 11% in the 2005–2008 cohort; similarly, they also found increasing contributing of NAFLD as cause for chronic liver disease rising from 47% to 76% for the earlier and later series, respectively [31]. These data are consistent with the clinical observation that NAFLD is now the commonest liver disorder seen in liver clinics of Western industrialised countries [4]. 

### 2.3. Age and the Prevalence and Severity of NAFLD

The prevalence of NAFLD in the general population increases with age; from 1 to 3% in children [32], 5% in teenagers [32, 33], 18% between 20 and 40 years, 39% in those aged 40 to 50 years, and to over 40% in those greater than 70 years [4, 26, 30, 34, 35]. In general, fatty liver is more prevalent in men than women up to the age of 60 years. Beyond menopause, the prevalence of fatty liver rises sharply in women and exceeds that observed in their male counterparts [5, 13, 36]. 

There are currently 2 epidemiological studies that have reported on fatty liver in individuals over 70 years. The first recruited 91 inpatients from 3 rehabilitation hospitals in Israel [34], and the second recruited 351 outpatients from a tertiary liver clinic in UK [37]. In the earlier study, Kagansky et al. adopted US and CT scan as diagnostic modality for NAFLD, while Frith et al. used liver biopsies. Both studies found NAFLD prevalence to exceed 40% in individuals over 70 years old. However, Frith et al. found a high prevalence of fibrosis (40%) and cirrhosis (14%) in the liver biopsies of these older individuals, which contradicted findings from Kagansky et al. who found no stigmata of chronic liver disease on clinical examination of their octogenarian cohort. The weakness of the Kagansky study was reliance on clinical examination for detection of advanced liver disease, which has very low sensitivity especially in the absence of decompensated cirrhosis [38].

### 2.4. Effects of Overweight, Insulin Resistance, and Impaired Glycemic Control on NAFLD Prevalence

The prevalence and severity of NAFLD is also influenced by presence of metabolic risk factors, such as overweight/obesity and T2D. The prevalence of NAFLD and NASH in T2D are 76% and 22%, respectively [27]. Furthermore, the prevalence of NAFLD correlates with the degree of impaired glucose metabolism, increasing from 27% in subjects with normal fasting glucose (fasting blood glucose, FBG < 6.1 mmol/L), to 43% and 62%, respectively, in those with impaired glucose tolerance (FBG ≥ 6.1 mmol, but < 7 mmol/L) and T2D (FBG > 7.0 mmol/L) [30]. Likewise, the prevalence of NAFLD increases in proportion to body weight category. In the Dionysos study, NAFLD was present in 24.5%, 67%, and 94% of the normal weight, overweight, and obese population, respectively [25, 39]. Pooled analysis of liver biopsy reports in bariatric surgery patients (BMI greater than 40 kg/m^2^ or greater than 35 kg/m^2^ for those with medical comorbidities) has shown steatosis and NASH prevalence to be 61% and 36%, respectively, whereas fibrosis and cirrhosis are present in 16% and 2%, respectively [4, 25, 26]. 

However, although NAFLD and NASH are more common in obese patients, it is now recognised that some of these patients do not meet the weight criteria for obesity. Not surprisingly, this is more common among Asian patients (even with ethnic-specific criteria), though it has also been increasingly recognised in Western Countries [4]. In China, for example, 40% of patients with NASH do not meet ethnicity-adjusted BMI for overweight or obesity [40, 41]. However, most patients with NAFLD/NASH whose BMI is within an ethnically adjusted “normal range” can be described as “metabolically obese,” where they have increased visceral fat tissue (VAT) and usually have detectable insulin resistance (IR) in spite of normal BMI [4, 36, 42].

## 3. Ageing Changes in Liver and Other Tissues Relevant to NAFLD/NASH 

Age-related cellular and organ system changes are not uniform. In this section, we focus on changes within the liver and the pattern of fat distribution, the latter with respect to metabolic characteristics that are relevant to NAFLD. 

### 3.1. The Ageing Liver

Between the ages of 20 and 70, there is a decline in hepatic blood flow (by 33%), hepatic volume (up to 25%), and liver function [43]. The impact of reduction of liver volume and hepatic blood flow in the elderly is unclear, but it tends to alter the pharmacokinetic profiles of drugs that undergo mandatory hepatic oxidation [44]. The octogenarian liver also has fewer, but larger, hepatocytes, increased polyploidy, and higher binuclear index [44], as well as a reduction in mitochondria numbers. The later may impact on oxidative respiration. Additionally, there is a reduction in bile acid synthesis, with consequent change to bile acid secretion and bile flow. There is an age-related decline in hepatic metabolism of LDL cholesterol, leading to elevated serum cholesterol. The combined effects of changes in bile acid secretion and cholesterol metabolism likely contribute to increased serum cholesterol levels and an increased frequency of gallstones formation. 

The ageing liver does appear to be more susceptible to the effects of drugs and other toxins, having diminished regenerative capacity to recover from insults, as evident by an increase in morbidity and mortality in hepatic resections in experimental studies in patients greater than 60 years old [44, 45]. 

### 3.2. Age-Related Changes in Body Composition, and Consequences for Metabolic Syndrome

There is an age-linked increase in abdominal adiposity and fat deposition in muscles (skeletal and cardiac), liver, and bone marrow [46]. The loss of lean body (muscle) mass is masked by the increase in total and regional adiposity with ageing. Not surprisingly, anthropometric indices such as body mass index (BMI) and waist circumference do not accurately reflect total and regional adiposity as well as they do in younger patients [46]. Also, the body fat distribution in the elderly is shifted from subcutaneous adipose tissue (SAT) to VAT locations, resulting in deleterious metabolic consequences such as IR [47, 48]. 

Several studies have now shown increasing prevalence of the metabolic syndrome with advancing age [49, 50]. Utilising data collected from 31,126 adult (>20 years) in NHANES group from 1999 to 2004, Churilla et al. showed an increasing likelihood of developing metabolic syndrome with age. Specifically, the odds ratio of developing metabolic syndrome rises from 1.66 to 5.93 in those aged 30–39 years to 60–69 years, respectively. Beyond this, the odds ratio declined slightly, to 4.39, in those over 80 years old [49]. 

As discussed before, there is a very strong correlation between metabolic syndrome and the subsequent development of NAFLD conversely, presence of hepatic steatosis is predictive for development of metabolic syndrome [13, 25, 36, 51]. Yamada et al. retrospectively looked at approximately 13,000 individuals undergoing routine health checkup and found that the incidence of T2D was 2.9% in men with fatty liver, compared with 0.6% in men without fatty liver. Similarly, T2D was found in 2.5% of women with fatty liver compared to 0.4% who did not develop fatty liver on ultrasound over the 5-years studied period [42]. In light of these and similar studies, it is not surprising that the age and gender specific prevalence trends for NAFLD mirror those for metabolic syndrome, peaking in middle age, and decreasing in octogenarians [13, 36, 37, 52]. 

## 4. Pathogenesis of NAFLD

The pathogenesis of NAFLD and progression to steatohepatitis have not been fully deciphered. In this section, we will briefly discuss what is known so far, with special reference to aspects relevant to interactions with ageing. 

An older concept of NASH pathogenesis, the so-called “two-hit” hypothesis of Day and James [58], proposed that hepatocyte TG accumulation resulting from metabolic imbalance (obesity, IR and diabetes) is what leads to steatosis (the “first hit”) and that the lipid-laden liver is then vulnerable to injurious processes (“second hit” insults) such as cytokines and oxidative stress [58, 59]. Damaged and dying hepatocytes and/or recruited and activated inflammatory cells, such as Kupffer cells, generate other signals (cytokines, growth factors, and oxidative stress) which activate scar-forming hepatic stellate cells with resultant development of liver fibrosis and cirrhosis [60, 61]. While this older concept has been useful for focusing attention on proinflammatory and profibrotic mechanisms in fatty liver disease, it does not account for why the majority of cases of simple steatosis do not progress to NASH or cirrhosis and it fails to take into account the cytotoxic and proinflammatory properties of several lipid species. Thus, a more encompassing concept about NASH pathogenesis considers that the profile of lipid molecules in NASH differs from that of simple steatosis, acknowledging that TG is a nontoxic safe storage form of lipid in tissues such as the liver but that other molecules (candidates include free fatty acids (FFA), toxic sphingophospholipids-like ceramide, diacylglycerides (DAG), free cholesterol, and oxysterol metabolites) could mediate tissue injury directly, a process termed “lipotoxicity” [62]. Lipotoxicity is also favoured as the major pathway for pancreatic beta cell injury in T2D [62–66] and as part of the process of atherogenesis and cardiac toxicity with metabolic syndrome [62]. 

### 4.1. Mechanism of Steatosis

**Figure 1:**
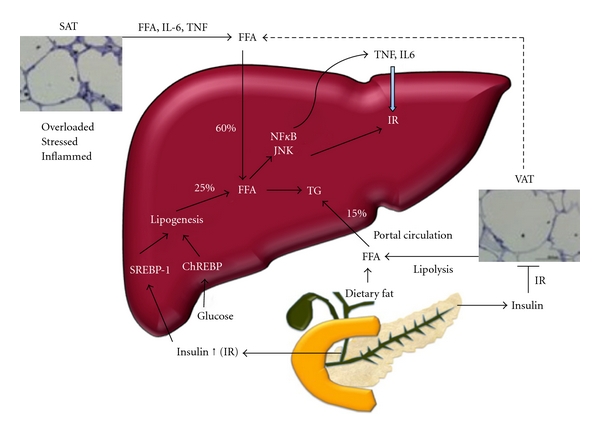
Mechanism of hepatic steatosis, adapted from Larter et al. 2010 [12], showing also interactions between adipose tissue in differing sites with liver in the development of insulin resistance (IR). FFA: free fatty acids; SAT: subcutaneous adipose tissue; VAT: visceral adipose tissue; TG: triglyceride.

Accumulation of fat in the liver represents an imbalance in hepatic lipid turnover. The liver plays a pivotal role in lipid metabolism. It takes up circulating free fatty acids and other lipids that arise from intestinal uptake/dietary sources, from lipolysis of peripheral storage sites (adipose tissue) and *de novo* synthesis (lipogenesis). The liver then exports the lipid for storage in adipose stores as triglyceride-rich very low density lipoproteins (VLDL). The mechanisms potentially contributing to hepatic steatosis are summarised in Figure 1.

Steatosis occurs when FFA supply to the liver (from dietary intake, peripheral lipolysis, and *de novo *lipogenesis) exceeds hepatic FFA elimination (via oxidation, re-esterification, and excretion as very low density lipoproteins (VLDL)). These pathways have recently been reviewed and will only be discussed briefly here [11, 12, 26, 67]. Kinetic studies indicate that approximately 75% of hepatic lipids (TAG/TG) in obese patients with NAFLD comes from peripheral sites (60% from nonesterified free fatty acids from lipolysis and 15% from diet), with approximately 25% arising from *de novo* lipogenesis [68]. The latter process is governed by several nuclear transcription factors that are activated by insulin (in the case of sterol regulatory element binding proteins (SREBP)1 and 2) and glucose (in the case of carbohydrate-responsive sterol regulatory element binding protein (ChREBP)1) [11, 12, 69]. Both SREBP1 and ChREBP activate fatty acid synthase (FAS), the rate limiting step in biosynthesis of long chain fatty acids, while SREBP2 regulates cholesterol biosynthesis. These pathways provide a partial explanation why insulin resistance and premetabolic syndrome (which is hyperinsulinemia and glucose intolerance) are strongly associated with steatosis. A more important contribution may come from dysregulation or “failure” of peripheral adipose tissue storage sites leading to store excess energy as TG, to abnormal lipid partitioning to the liver and other nonphysiological storage sites like muscles [12]. 

Several studies have clearly indicate that the development of NAFLD and of metabolic syndrome is more closely linked to the pattern of fat distribution than to total body fat. In particular, central (or visceral) adiposity is strongly implicated in the development of both hepatic steatosis and metabolic syndrome [17, 60, 70]. Clinical studies have also highlighted the adverse contribution of visceral adipose tissue (VAT) to the metabolic and liver complications of overweight/obesity [48, 71]. Similarly, in *ob/ob *(leptin deficient) mice, which are hyperphagic, develop insulin resistance and have severe steatosis [48], an adiponectin transgene (which restored normal serum adiponectin levels) expanded subcutaneous adipose and worsened obesity, but improved metabolic indices, such as glycemic control, in association with amelioration of NAFLD [72]. In other murine experiments, using C57B/6 mice, Tran et al. found transplanting subcutaneous adipose tissue (SAT) to a VAT location, or to a lesser extent to another SAT location, improved insulin sensitivity and reduced body weight of recipient animals. This indicates possible intrinsic physiological difference in the adipocytes between the two sites [47]. 

The deleterious metabolic effect of increased VAT can be partially explained by dysregulation of adipocytokines (TNF-*α*, leptin, resistin, and most notably adiponectin). This results from increased recruitment of inflammatory cells, particularly macrophages [73] in the setting of stressed and hypertrophic adipocytes caused by overnutrition and obesity [74]. VAT secretes more proinflammatory cytokines (TNF-*α*, IL-6, and monocyte chemoattractant protein-1 (MCP1)), and this, coupled with direct drainage to the liver via the portal circulation, emphasizes the ability of VAT to directly impair hepatic insulin signaling and promote inflammation. TNF-*α* can activate both nuclear factor-kappa B (NF*κ*B) and c-jun *N*-terminal kinase (JNK), promoting serine phosphorylation of the insulin receptor substrate which directly impairs insulin signalling. Additionally, MCP-1 can activate inflammatory pathways and promote hepatocyte TG accumulation directly. 

Insulin resistance in adipose tissue allows inappropriately sustained lipolysis with release of FFA, which are shunted to the liver at times when the liver is programmed for lipogenesis rather than for fat disposal [66]. Adipose inflammation, coupled with hepatic insulin resistance, is one of many possible connections linking adipocytes and liver in NASH, as addressed next [12]. 

The ageing process results in increased prevalence of metabolic syndrome and T2D, possibly via preferential fat distribution to VAT sites. These factors all culminate in dysregulated lipid handling by the liver, causing steatosis and partially explaining the resultant progression of NAFLD to NASH. 

### 4.2. NAFLD and Insulin Resistance

Insulin resistance is found in virtually every patient with NASH and in approximately 60% of all patients with NAFLD. The pathogenesis of insulin resistance involves a combination of genetic polymorphisms that influence insulin secretion and many acquired factors, such as sedentary lifestyle, medications, chronic illnesses, ageing, and other environmental factors which promote obesity and immobility [26, 78]. Insulin resistance, whether acquired or genetically determined, raises serum insulin and increases serum free fatty acid (FFA) levels. In the presence of a steatotic liver, the hyperinsulinemic state fails to suppress adipose FFA flux, resulting in these FFA being taken up by the liver, driving TG production and ultimately perpetuating more hepatic steatosis and inflammation when the mechanisms for lipid storage in adipocytes become overwhelmed [10]. As discussed earlier, the accompanying hyperinsulinemia promotes *de novo* hepatic lipogenesis [79], further promoting lipid overload in hepatocytes.

### 4.3. What Drives Progression to Steatohepatitis (NASH) Once Steatosis Occurs?

Significant hepatocyte apoptosis is a feature of NASH and forms the basis of a serum test for caspase3-generated cytokeratin-18 fragments (a biomarker of apoptosis), which is being increasingly used to differentiate between patients with NASH and those with simple steatosis [26, 62, 80]. Hepatocyte apoptosis itself triggers regenerative mechanisms to replace dead hepatocytes. However, aberrant repair in some individuals eventually leads to activation of hepatic stellate cells (HSC) to myofibroblasts and hepatic recruitment of proinflammatory and profibrogenic immune cells to the liver [61]. 

As liver injury and cell death progresses, fat laden hepatocytes and perisinusoidal fibrosis may impair microvascular hepatic blood flow, causing decreased oxygen and nutrient exchange, and thereby stimulating microvascular inflammatory response and a self-perpetuating cycle of liver damage and vascular insufficiency [26, 81]. 

These self-perpetuating cycles of inflammation and apoptosis are effected in some ways by adipokines, toxic lipid species, mitochondrial dysfunction, vascular disturbance, and possibly gut bacterial endotoxins [26]. Ageing can alter some of these modulators, such as by changes in SAT/VAT distribution with its effect on adiponectin levels [82], reduced liver blood flow, and reduced ability of ageing liver to adapt to injury. Such changes could contribute to worsened liver histology of NAFLD in older people.

## 5. Progression of NAFLD: Lessons from Natural History Studies

Although simple steatosis is generally nonprogressive [83], the initial assessment of SS as always being benign is not fully supported by current evidence [53–56]. Documentation of progressive fibrosis is problematic because serial liver biopsies are necessary and there remains the possibility of sampling error.  Table 1 summarizes 4 early studies using serial liver biopsies to assess disease activity and progression in NAFLD/NASH. In essence, the studies have shown progression in disease activity (SS to NASH, NASH to fibrosis, and fibrosis to cirrhosis) in up to a third (30–37%) of patients, while a quarter (23–29%) shows the reverse, that is, histological improvement. Both obesity and BMI were predictive of disease progression [55, 56]. Additionally, more severe grades of baseline steatosis, elevated serum alanine aminotransferase (ALT), platelet count, and weight gain greater than 5 kg were other predictors of disease progression [53, 54]. However, none of these studies looked at patients greater than 70 years old.

What is clear in NAFLD is that once advanced fibrosis has developed, the risk for hepatocellular carcinoma (HCC) is about 5–7%. If the person is cirrhotic at time of diagnosis, the risk of developing portal hypertension as a major complication is also high; 17%, 23%, and 52% at 1, 3, and 10 years, respectively [84]. 

Many cross-sectional studies have sought predictors of hepatic fibrosis in NAFLD. Relevant factors include age (especially over age 50 years), BMI > 28–32 kg/m^2^, insulin resistance or T2D, and raised serum ALT [13, 14, 27, 57, 85–91]. In multivariate analysis, age, BMI, arterial hypertension, ALT, insulin resistance, and hepatic necroinflammatory grade were shown to independently predict presence of fibrosis [27, 36, 57, 86, 87, 89, 91]. 

The other key aspect of clinical outcome studies in NAFLD is the increased likelihood of death from cardiovascular diseases (coronary heart disease, stroke) and nonhepatic malignancy. However, liver-related mortality ranks third in the causes of death. The risk of liver-related death is even higher in the subgroup of patients with NASH compared to those with SS (2–10% for NASH versus 0–2% for SS) (Table 2) [57, 75–77]. 

## 6. Management of NAFLD in the Elderly

The cornerstone of the management of NAFLD is to correct the disturbed metabolic milieu by encouraging an active lifestyle so as to counteract increases in body weight and improve insulin sensitivity. Further, treatment of coexisting metabolic disorders like hypertension, dyslipidaemia, and glucose intolerance/diabetes is important in the overall management plan. 

### 6.1. Lifestyle Changes

While lifestyle changes are widely promoted, adherence remains a major issue. A program of cognitive behavioural therapy remains the most effective tool to obtain long-term adherence. In these programs, individuals are educated to self-manage their diet and to undertake moderate daily physical activity [92, 93]. Compliance to modest caloric restriction and increased physical activity result in both sustained weight loss and improved cardiorespiratory fitness, the latter assessed by the peak oxygen consumption (VO_2_ max). In one study, a 21% improvement in physical performance measures was observed in the diet-exercise group as compared to 12% and 15% in the diet alone and exercise alone groups, respectively [94]. 

Weight loss of only 5–10% (e.g., 3.7 kg in a 75 kg man) decreases liver fat by 40% in both nondiabetic and diabetic subjects [60, 95]. Moreover, aerobic exercise reduces hepatic fat content independent of weight loss [96]. The beneficial effects on hepatic steatosis are likely a consequence of increased insulin sensitivity through reduced peripheral lipolysis, inhibition of lipid synthesis, and stimulation of FA oxidation [97]. On the other hand, any effects on hepatic inflammation and fibrosis have been inconclusive [98]. In individuals aged over 65 years, the beneficial effect of diet and exercise on physical fitness, muscle strength [99], and *metabolic* fitness as shown by reduction in hepatic steatosis, serum cholesterol, high blood pressure, and improved insulin sensitivity Shah et al. [99] have all been confirmed. Further studies on the efficacy of such interventions in clinical and histological outcomes in NAFLD/NASH will be of interest.

### 6.2. Pharmacotherapy—The Insulin Sensitisers

Currently, there are no approved drugs for use in the treatment of fatty liver or NASH. As insulin resistance is central to NAFLD, agents that improve insulin sensitivity appear promising. To date, two classes of insulin sensitising agents, metformin, and thiazolidinediones (TZDs) have been evaluated. 

Metformin causes weight reduction and improves insulin sensitivity by decreasing hepatic glucose output and increasing peripheral glucose uptake, reducing hepatic lipogenesis and increasing hepatic fatty acid *β*-oxidation (by activation of AMP-activated protein kinase), and suppression of lipogenic transcription factor, SREBP-1 [100]. Early studies of metformin in NASH showed significant reduction in hepatic steatosis and ALT, but histologic follow-up data are scarce and have not shown improvement in hepatic necroinflammatory grades [101–103], except in one study where it correlated with the degree of weight loss [101]. The use of metformin in the elderly could be also problematic because of the additional risk of severe life-threatening lactic acidosis (5 per 100,000 prescriptions) [104]. Currently, metformin use for NASH is not recommended [100, 105].

Thiazolidinediones are PPAR-*γ* agonists that exert insulin-sensitizing actions on adipocytes and in the liver. PPAR-*γ* agonists increase adipocyte numbers, promote their differentiation, and facilitate uptake and storage of FFA, thereby reducing ectopic fat deposition in liver and muscles and restoring insulin sensitivity. TZDs also increase serum adiponectin levels, with improved insulin sensitivity. In NAFLD, the TZDs have been shown to reduce serum ALT levels and hepatic steatosis, with some effects on necroinflammation activity, but effects on hepatic fibrosis have not usually been observed; this could relate to short duration of use, typically 6–12 months [106–109]. More recently, Ratziu et al. extended duration of rosiglitazone for an additional 2 years in 53 patients with liver biopsy-proven NASH. Disappointingly, even though rosiglitazone substantially improved steatosis in the first year, longer treatment did not improve NASH histology despite maintained effects on insulin sensitivity and ALT levels [110]. In general, TZDs used to treat NASH have been well tolerated, but significant weight gain (2–6 kg) predominantly in the SAT area occurs in up to 72% of patients and this is a concern. Also, with regards to the older patient with NAFLD, TZDs can precipitate heart failure and their use is not recommended for patients with New York Heart Association Class III and IV heart failure [67, 111]. Rosiglitazone has been withdrawn from the European market due to increased rates of myocardial infarction, but not overall cardiovascular mortality in a meta-analysis of approximately 35,000 patients from 56 randomised controlled trials [112]. Its access is restricted in Australia and America [113]. Pioglitazone appears to be the safer alternative. Troglitazone has been withdrawn worldwide due to significant fatal hepatotoxicity. 

### 6.3. Bariatric Surgery

Bariatric surgery is currently recommended for morbidly obese patients (BMI > 40 kg/m^2^) and for metabolic syndrome or T2D when BMI exceeds 35 kg/m^2^ [114]. Earlier approaches to obesity included jejunoileal bypass surgery, which was abandoned owing to unacceptable risks of liver failure. All other types of bariatric surgical techniques can decrease excess body weight by up to 50%, with generally less weight loss in laparoscopic procedures versus more invasive approaches [115–118]. This weight loss improves insulin sensitivity and reduces the frequency and severity of metabolic syndrome, diabetes and its complications [97]. With respect to NAFLD/NASH, follow-up liver biopsies have shown a reduction in hepatic necroinflammatory activity including resolution of NASH in the majority of cases and reduced hepatic fibrosis in some [119, 120]. Bariatric surgery in patients aged greater than 60 years old is associated with significantly increased overall morbidity (19%) compared with those less than 60 years (11%). However, with the exception of a small subset of patients with significant heart disease, there is no significant difference in the observed-to-expected mortality ratio [121]. Therefore, bariatric surgery can still be considered reasonable in carefully selected older patients, given its significant metabolic benefits.

### 6.4. Is There a Role for Liver Transplantation for NASH Cirrhosis in the Elderly?

Currently, 5–10% of liver transplants in the USA are for patients with NASH-related cirrhosis, while NASH is becoming an important predisposing factor for HCC. Because liver transplantation for hepatitis C peaked at 28% in 2002 and has remained stable since [31], it has been projected that NAFLD will become the most common indication for liver transplantation in the next 20–30 years [122]. The obesity epidemic affects not only recipients, but also potential organ donors, up to a quarter of whom have hepatic steatosis. The prevalence of steatosis in donor livers increases with BMI and age. Fatty change in the donor liver is associated with higher rates of primary graft nonfunction, likely due to the increased severity of hepatic ischemia-reperfusion injury [81]. 

Optimizing body weight before transplantation is rarely achieved and may be hazardous in patients with decompensated cirrhosis. Additionally, morbidly obese patients carry a high perioperative risk and are at increased risk for recurrence of progressive fatty liver disease after transplantation, especially in the setting of immunosuppressive therapy; hepatic steatosis occurs in up to 60% of transplant recipients, with 5–10% progressing to cirrhosis and graft loss [122]. Finally, metabolic syndrome is among the most common causes of death after all liver transplants and is likely to be accelerated among patients with NASH as the original cause of endstage liver disease.

The mean age for liver transplantation increased from 29 in 1985, to 41 in 1995. By 1999, 21% of liver transplant recipients were in people aged 60 years or older [123, 124]. Studies have also shown that, with careful selection, there are no differences in survival or length of hospital stay for individuals over 60 years old compared to their younger counterparts [124]. Therefore, liver transplantation may be a possible option in carefully selected patients with NASH cirrhosis. 

## 7. Conclusions

Disease incidence, severity, and progression in NAFLD/NASH are strongly associated with presence of components of metabolic syndrome, in particular diabetes and obesity, and are also governed by the interactive effects of both genetic background, sex, age, and environmental factors (food intake, level of physical activity). Advanced age is associated with disease severity and fibrosis progression; a relatively high proportion of individuals with progressive forms of NAFLD develop cirrhosis by the time there are in their 70s or beyond, although more data are required on the exact risks. Advanced forms of NAFLD (NASH and cirrhosis) are associated with increased standardised mortality and a relatively high risk of liver-related deaths. 

NASH is associated with increased risk of death from cardiovascular disease and nonliver malignancies, as well as from liver complications. The practical implication is that clinicians need to consider early interventions to optimise the management of modifiable metabolic risk factors, like glycemic control in T2D, hypertension, and dyslipidemia, each of which could also contribute to disease progression in NAFLD. For all patients with NAFLD, the cornerstone to management remains correction of modifiable risk factors. Exercise and dietary restriction can be very effective in carefully selected patients and should be used in a multidisciplinary approach, involving physiotherapists, dieticians, and occupational therapists to overcome potential physical limitations in older patients, such as osteoarthritis or decreasing mobility from other causes. The insulin sensitisers are not approved for use in this country for treatment of NASH, and they should be used with caution in elderly people given the increased likelihood of coexisting medical conditions like congestive heart failure. 

Decompensated NASH cirrhosis may become the number one indication for liver transplantation in the next two decades. This means that patients being considered for transplantation are likely to be much older and have longstanding medical comorbidities related to metabolic syndrome. These need to be assessed and managed prior to acceptance onto a transplantation list. Transplant physicians may need to manage weight loss prior to transplantation of the obese and morbidly obese, but also will be required to manage diabetes, osteoporosis, hypertension, dyslipidemia, immunosuppression, and the challenge of polypharmacy from prescriptions these elderly population will inevitably require.

## Figures and Tables

**Table 1:** Disease progression in NAFLD/NASH and risk factors predicting disease progression [53–56].

	Powell et al. 1990 [53]	Harrison et al. 2003 [54]	Fassio et al. 2004 [55]	Adams et al. 2005 [56]
Number of patients with serial liver biopsy	13	22	22	103
Age (years)	49 (16–70)	50.6 (33–64)	45 (20–69)	45 (19–65)
Biopsy interval (years)	1–9	N/A	1–3	0.7–21
Follow-up period (years)	1.5–21.5	1.4–15.7	3–14.3	N/A
Disease activity (%)				
Unchanged	46%	50%	68%	34%
Progress	30%	32%	31.8%	37%
Regress	23%	18%	0%	29%
Risk factors associated with NASH progression	None identified	AST	Obesity	Obesity
			BMI	BMI
				Low initial fibrosis score

N/A: not available; AST: aspartate transaminase.

**Table 2:** Natural history data on NAFLD.

	Adams et al. 2005 [57]	Ekstedt et al. 2006 [75]		Ong et al. 2008 [76]	Rafiq et al. 2009 [77]	
	NAFLD	NASH	NNFL	NAFLD	NASH	NNFL
*N*	435	71	58	817	72	101
Age at diagnosis (in years)	49 ± 15	55 ± 12	47 ± 12	17+	51 ± 13	49 ± 15
Males/females	213/222				30/70	47/53
Study period	1980–2000	1988–1993		1988–1994	1979–1987	
Followup (in years)	7.6 ± 4	13.7 ± 1.3		8.4 (median)	10.5 (median)	13.0 (median)
Advanced cirrhosis	13 (3.1%)	7 (9.8%)	0			
HCC	2 (0.5%)	2 (2.8%)	0			
Deaths (total)	53 (12.1%)	26 (20.3%)		80 (9.7%)		
IHD	13 (2.9%)	11 (15.5%)	5 (8.6%)	20 (2.4%)	7 (12.3%)	15 (20.3%)
Non-HCC cancer	15 (3.4%)	4 (5.6%)	1 (1.7%)	19 (2.3%)	5 (8.8%)	9 (12.2%)
Liver*	7 (1.6%)	2 (2.8%)	0	5 (0.6%)	10 (17.5%)	2 (2.7%)

NNLF: non-NASH fatty liver (which equates with simple steatosis referred to in the text); *liver related mortality.
